# Microwave characterisation of carbon nanotube powders

**DOI:** 10.1186/1556-276X-7-429

**Published:** 2012-08-01

**Authors:** Adrian Porch, Daniel I Odili, Peter A Childs

**Affiliations:** 1 School of Engineering, Cardiff University, Newport Road, Cardiff, CF24 3AA, UK; 2 School of Electronic, Electrical and Computer Engineering, University of Birmingham, Pritchatts Road, Birmingham, B15 2TT, UK

**Keywords:** 73.63.Fg, 72.80.Rj, 78.70.Gq, Carbon nanotubes, Microwave, Screening

## Abstract

We have used a 3-GHz microwave host cavity to study the remarkable electronic properties of metallic, single-walled carbon nanotubes. Powder samples are placed in its magnetic field antinode, which induces microwave currents without the need for electrical contacts. Samples are shown to screen effectively the microwave magnetic field, implying an extremely low value of sheet resistance (< 10 μΩ) within the graphene sheets making up the curved nanotube walls. Associated microwave losses are large due to the large surface area, and also point to a similar, very small value of sheet resistance due to the inherent ballistic electron transport.

## Background

There has been intensive research on the characterisation and application of carbon nanotubes for next-generation electronic devices [1]. Ballistic conduction along single-walled metallic nanotubes is predicted theoretically and has been experimentally verified. Clearly, this has enormous impact for electronic devices fabricated using these remarkable materials, but there are problems in characterising the electronic properties using conventional (i.e. DC) techniques. This is mainly due to the need to make electrical contacts with the ends of the nanotube, which suppresses the measured conductivity, and is further complicated by inter-tube contacts. For example, it has been found that thin film samples have sheet resistances limited by the large junction resistances between adjacent nanotubes [2].

In this context, we propose a new method to characterise the electronic properties of metallic nanotubes using microwaves as a means of exciting currents, thus negating the requirement for electrical contacts. The method is fast and is performed on a powder sample, so it allows rapid measurements of large numbers of samples and permits *in situ* changes in electronic properties (e.g. due to surface adsorption of chemical species) without the need to remove the sample from the apparatus. Previous microwave studies of carbon nanotube materials have ranged from the screening efficiency of thin films [3] to the microwave excitation of single nanotubes [4]. In our experiments, powder samples are placed in a region of high microwave magnetic field in a microwave cavity (here, a 3-GHz copper hairpin), and the sample properties are determined using the cavity perturbation technique. We have used a similar method to measure the electronic properties of a range of other material systems (e.g. metal-loaded zeolites [5]).

## Methods

**Figure 1:**
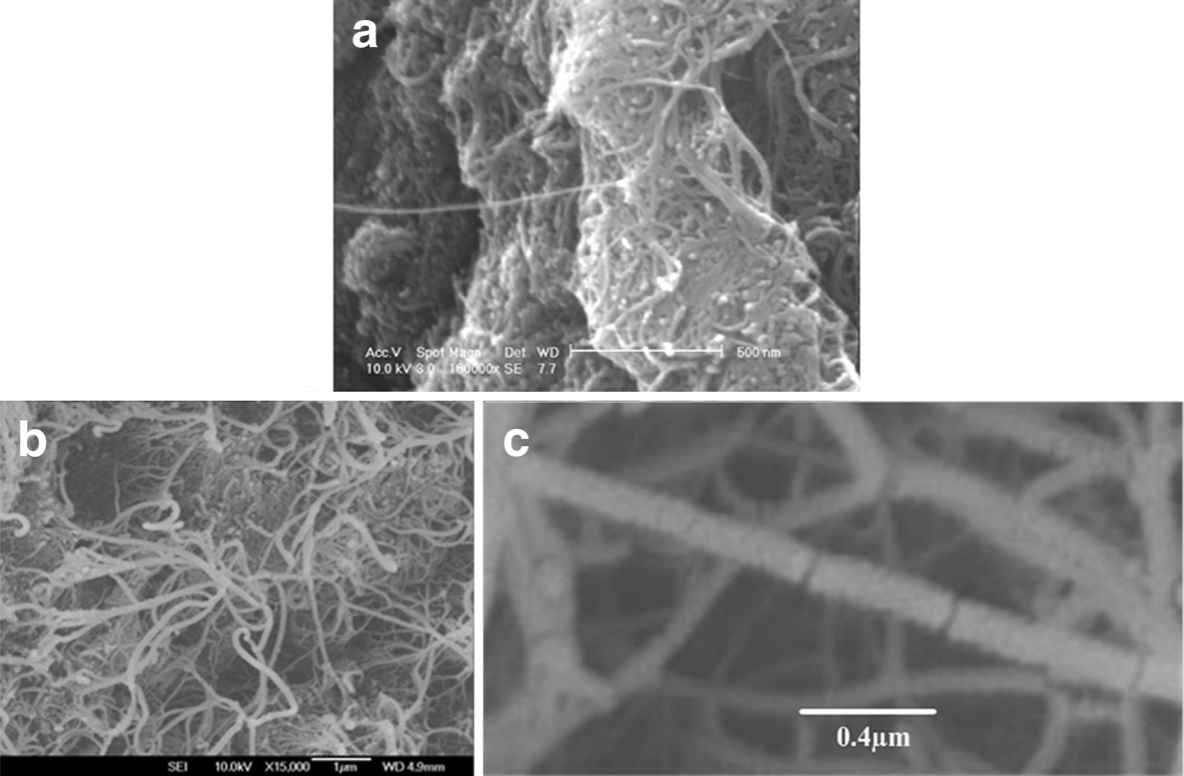
**SEM image of the nanotubes used in this experiment.** (**a**) Before and (**b**,**c**) after manual grinding.

Powdered nanotube samples were obtained from Thomas Swan & Co. Ltd. (County Durham, UK), prepared by thermal CVD at 800°C, giving a mix of semiconducting and metallic single-walled nanotubes. Figure 1 shows the scanning electron microscope (SEM) image of the nanotubes before and after manual grinding. The long nanotubes are broken into smaller segments confirming the production of short carbon nanotubes, which has been previously reported using the ball milling technique.

**Figure 2:**
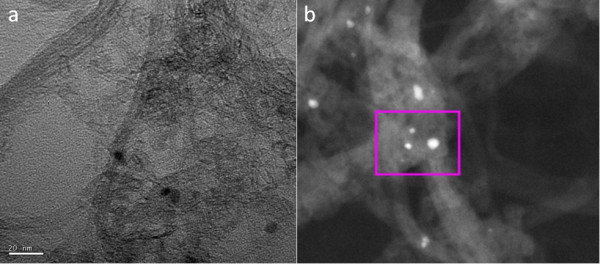
**TEM and STEM images of nanotubes.** (**a**) TEM image of the nanotubes; dark spots (impurities). (**b**) STEM image; evidence of Fe content (inset).

**Figure 3:**
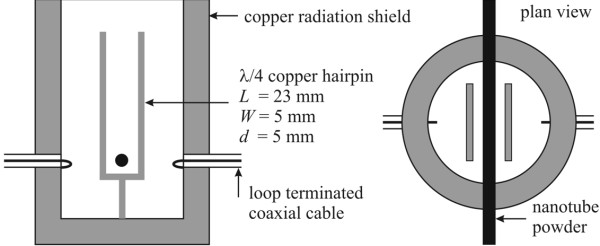
**Schematic diagram of the copper hairpin resonator.** Samples are placed at the magnetic field antinode shown with *H* parallel to the axis of the quartz tube holding the powder sample. *L*, length; *W*, width; *d*, diameter.

Analysis of the transmission electron microscope (TEM) images shown in Figure 2 suggests the nanotubes have diameters ranging from 1.2 to 1.7 nm and lengths of 100 nm to 4 μm. Scanning transmission microscopy (STEM) of the nanotubes shows that there are iron (Fe) impurities on the surface of the nanotubes. Energy dispersive X-ray spectroscopy gives atomic percent ratios of 96.5:2.7:0.8 for C:O:Fe, respectively. Samples were loaded into low-loss quartz tubes (inner diameter of 1.3 mm and wall thickness 0.35 mm) and placed parallel to the microwave magnetic field in the 3-GHz quarter-wave copper hairpin cavity, shown in Figure 3.

The hairpin itself is a 1-mm thick copper strip, with a width of 5 mm, bent into a U shape of length 23 mm and plate separation of 5 mm. The expected resonant frequency of the fundamental mode of the hairpin is, therefore, *f*_0_ ≈ *c*/4 *L* ≈ 3.26 GHz, in practise about 10% lower due to the end effects of electric and magnetic fields spilling outside of the structure. The microwave magnetic field is largest at the short-circuit end of the hairpin, where the field is also approximately uniform and runs parallel to the plate surfaces. The sample is inserted into this region. The hairpin is surrounded by a cylindrical radiation shield, of length twice that of the hairpin (i.e. 50 mm) and inner radius of 15 mm. This reduces the radiation losses from the hairpin and, thus, preserves a high quality factor, which is desirable for measuring the additional microwave loss contributed by the sample. Microwave power is coupled in and out of the hairpin using an identical pair of loop-terminated RG405 microwave coaxial cables which couple to the microwave magnetic field at the hairpin’s short-circuit end. The transmitted microwave power (i.e. |*S*_21_|^2^) is measured in the frequency domain using an Agilent E5071B network analyser with computer control (Agilent Technologies, Inc., CA, USA). The unloaded quality factor *Q* of the empty cavity is around 1,500, i.e. large for its small volume (≈ 600 mm^3^), thus enhancing the sample filling factor for more sensitive measurements of its electronic properties. Cavity couplings are kept weak (i.e. insertion loss > 30 dB at resonance), so that only minor correction factors are needed to extract unloaded *Q* factors from the raw data.

## Results and discussion

**Figure 4:**
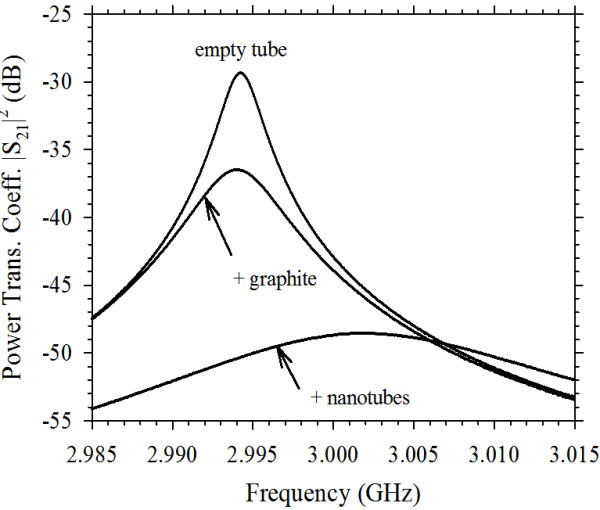
**Resonant traces for the nanotube sample compared with graphite power.** The increased resonant frequency of the nanotube sample is due to effective screening of the *H*-field.

Microwave results for a nanotube sample are shown in Figure 4, compared with a similar volume of graphite powder. Remarkably, this nanotube sample exhibits screening of the microwave magnetic field. This is evidenced by an increased resonant frequency of Δ*f*_0_ = 9.0 ± 0.3 MHz relative to the empty tube. There are also very large microwave losses, evidenced by an increased 3-dB bandwidth of Δ*f*_B_ = 18 ± 1 MHz. Such screening is usually observed only in powdered (or bulk) metals and of conductivity large enough for the mean particle size to become much greater than the microwave skin depth. As can be seen from the data of Figure 4, the conductivity of graphite is not high enough to observe any such screening; rather, it exhibits the response typical of a lossy dielectric (i.e. a broadened 3-dB bandwidth with no frequency shift, indicative of full sample penetration by the microwave magnetic field).

**Figure 5:**
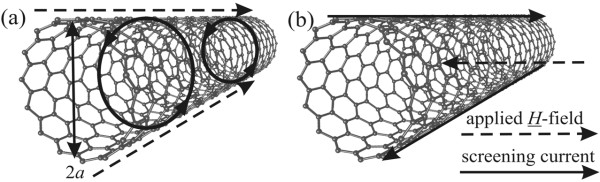
**Screening currents for the microwave-field.** Applied (**a**) parallel and (**b**) perpendicular to the nanotube axis.

To analyse this data, we use standard cavity perturbation theory, which assumes a small sample filling fraction within the cavity [6]. For the *H*-field applied parallel to the nanotube axis (Figure 5a), the main results are the following:

(1) $$\frac{\Delta f_{0}}{f_{0}}\approx -\frac{\omega^{2}\tau^{2}}{1+\omega^{2}\tau^{2}}\frac{V_{\text{s}}}{2V_{\text{eff}}}$$

(2) $$\frac{\Delta f_{\text{B}}}{f_{0}}\approx \frac{\omega \tau }{1+\omega^{2}\tau^{2}}\frac{V_{\text{s}}}{V_{\text{eff}}}$$

where *V*_s_ is the total sample volume; *V*_eff_, effective cavity volume; and *τ*, relaxation time, defined via

(3) $$V_{\text{eff}}=\int_{\text{cavity}}{\left(H/H_{0}\right)}^{2}dV\approx LWd/2\text{,}\;\tau =\mu_{0}a/2R_{\text{sq}}$$

with *a* being the average nanotube radius, *H*_0_ the local magnetic field strength at the sample and *R*_sq_ the sheet resistance of graphene walls of the nanotubes. As can be seen from Figure 5, both axial and radial components of *H* relative to the nanotube axis will be screened if the sheet resistance is low enough. Effective screening only occurs in the limit when *ωτ* > 1, i.e. when *R*_sq_ < μ_0_*a*ω/2.

A separate calibration experiment involved the insertion of small copper spheres of radii 330 μm into the same *H*-field position as for the sample. The observed frequency increase of Δ*f*_0_ = 1.30 ± 0.01 MHz per sphere allows us to determine experimentally *V*_eff_ = 270 ± 2 mm^3^, comparing well with the expected value of *LWd*/2 ≈ 290 mm^3^. Thus, the observed nanotube frequency shift corresponds to an effective screened volume of ≈ 1.7 mm^3^.

## Conclusions

The inner tube volume within the cavity is ≈ 6.6 mm^3^, of which ≈ 0.40 mm^3^ is nanotube material (i.e. a packing fraction of about 6% by volume, based on the sample mass of 1.3 mg). From this, we conclude that the magnetic field is effectively screened within this low density powder, both from within the nanotubes themselves and from a much larger volume (×3) in the space outside. For this to happen, we also conclude that the nanotube sheet resistance must be extremely small, i.e. an upper limit of *R*_sq_ ≈ 10 μΩ based on a mean nanotube radius of *a* ≈ 0.7 nm. Since a trace of metallic Fe is present in the sample, it is prudent to quantify how much this contributes to the microwave screening. The 0.8% atomic ratio of Fe atoms corresponds to a volume of about 0.006 mm^3^. This is likely to be dispersed as very small particles of radii less than 1 μm. Given that the skin depth of Fe at 3 GHz at room temperature is about 2.8 μm, we would not expect such small particles to screen the microwave magnetic field. Even if the Fe formed a single large particle, which gives rise to the greatest screened volume, the contribution to increase in resonant frequency will be only 0.6% of that observed for the whole CNT sample. Hence, we conclude that the screening effect of the Fe is negligible.

Does such a small value of *R*_sq_ contradict the observation of the large microwave losses of Figure 4? The answer can be found in the huge surface area of carbon nanotube powders, e.g. our 1.3-mg sample has a total surface area *S* ≈ 1.1 m^3^. Sheet resistance can be extracted from Equation 1b in the limit of strong screening *ωτ* > > 1, resulting in *R*_sq_ ≈ 2πμ_0_*V*_eff_ Δ*f*_B_ / *S* ≈ 30 μΩ. Therefore, these two independent measurements (one of microwave screening, the other of microwave loss) both point to very small values of sheet resistance due to ballistic transport. In experimental studies of carbon nanotube films, the sheet resistance is found to be of the order of 1 kΩ [7] with similar results for graphene films [8]. In these studies, the intrinsic sheet resistance of the carbon nanotubes can be difficult to extract from the measurements as charge transport is limited by contacts between the nanotubes. Other types of measurement such as the optical conductivities reported in [8] do not circumvent this problem as they characterise relatively large areas of the films. In theoretical studies of devices based on discrete carbon nanotubes and of carbon nanotube films, charge transport is often assumed to be ballistic, and therefore, the sheet resistance is zero. Ballistic transport has been observed in ohmically contacted metallic single wall carbon nanotubes having lengths less than approximately 300 nm [9].

Not all of the nanotube powders studied to date show such striking behaviour, so future experiments will concentrate on systematic studies of a wider range of materials (semiconducting and metallic), over a wider range of frequencies (particularly from kilohertz to megahertz), also trying to identify the nature of the defects giving rise to the finite sheet resistance. Indeed, isolating metallic samples is, itself, an important problem, and the method we have proposed here may serve as a means of quantifying the volume fraction of metallic nanotubes within a given powder.

## Competing interests

The authors declare that they have no competing interests.

## Authors’ contributions

AP and DIO carried out the microwave measurements, and DIO characterized the sample. AP and PAC conceived of the study and completed the analysis. All authors read and approved the final manuscript.
